# Attitudes of Patients with Cancer towards Vaccinations—Results of Online Survey with Special Focus on the Vaccination against COVID-19

**DOI:** 10.3390/vaccines9050411

**Published:** 2021-04-21

**Authors:** Anna Brodziak, Dawid Sigorski, Małgorzata Osmola, Michał Wilk, Angelika Gawlik-Urban, Joanna Kiszka, Katarzyna Machulska-Ciuraj, Paweł Sobczuk

**Affiliations:** 1 Laboratory of Centre for Preclinical Research, Department of Experimental and Clinical Physiology, Medical University of Warsaw, 02-097 Warsaw, Poland; anna.brodziak@wum.edu.pl; 2 Department of Oncology and Radiotherapy, Maria Sklodowska-Curie National Research Institute of Oncology, 02-781 Warsaw, Poland; 3 Department of Oncology, University of Warmia and Mazury in Olsztyn, 11-228 Olsztyn, Poland; dawidsigorski@wp.pl; 4 Department of Oncology and Immuno-Oncology, Warmian-Masurian Cancer Center of the Ministry of the Interior and Administration’s Hospital, 11-228 Olsztyn, Poland; 5 Department of Hematology, Transplantation and Internal Medicine, University Clinical Centre, Medical University of Warsaw, 02-097 Warsaw, Poland; malgorzata.osmola@wum.edu.pl; 6 Centre of Postgraduate Medical Education, Department of Oncology, European Health Centre, 05-400 Otwock, Poland; wilk.m@onet.eu; 7 Department of Clinical Oncology, Maria Sklodowska-Curie National Research Institute of Oncology, 31-115 Kraków, Poland; a_gawlik_urban@pwsztar.edu.pl; 8 Subcarpathian Cancer Center, 35-055 Rzeszow, Poland; kiszka_joanna@wp.pl; 9 Department of Clinical Oncology, Saint John of Dukla Oncology Centre of the Lublin Region, 20-090 Lublin, Poland; k.machulska@interia.pl; 10 Department of Soft Tissue/Bone Sarcoma and Melanoma, Maria Sklodowska-Curie National Research Institute of Oncology, 02-781 Warsaw, Poland

**Keywords:** vaccination, vaccine, cancer, COVID-19, influenza

## Abstract

Recently developed COVID-19 vaccines significantly reduce the risk of severe coronavirus disease, which is essential in the particularly vulnerable cancer patient population. There is a growing anti-vaccine concern that may affect the success of the fight against the SARS-CoV2 pandemic. To evaluate opinions and attitudes toward vaccination, we conducted an anonymous online survey among Polish patients diagnosed with cancer. We analyzed how socio-demographic factors, type of cancer, comorbidities, previous influenza vaccinations, and information sources affect the general willingness and opinions about vaccinations, emphasizing vaccination against COVID-19. Six hundred thirty-five patients (80.2% female) participated in the study. A positive attitude towards vaccination was presented by 73.7%, neutral by 17.8%, while negative by 8.5%. Willingness to get vaccinated was declared by 60.3%, 23.5% were unwilling, and 16.2% were undecided. Significant predictors of willingness were education, marital status, active anti-cancer treatment, previous influenza vaccination, and positive attitude towards vaccinations. Patients with cancer have concerns regarding safety, effectiveness, and the process of development of the COVID-19 vaccine. Overall, patients with cancer present positive attitudes towards COVID-19 vaccination but required sufficient information on its efficacy and side effects.

## 1. Introduction

Infectious diseases have been present throughout human history and for many years were the main reason for death. Several pandemics, such as the Spanish Flu and the Black Death resulted in millions of casualties [1]. With the first vaccination against smallpox developed in 1796 by Edward Jenner, constant progress in this area has been observed [2]. Smallpox, caused by the variola virus, is the only human infectious disease to have ever been eradicated worldwide; however, several infectious diseases like polio, measles, rubella have been curtailed thanks to vaccination efforts. Despite advances in vaccination science, several viral infections like HIV and HCV are still a significant epidemiological problem [3]. Vaccinations allow the reduction of morbidity and mortality, which is especially important in vulnerable populations. One such population is cancer patients, who are at high risk of severe complications and death from infection. This is caused by the presence of many risk factors such as immune suppression, advanced age, comorbidities and malnutrition [4]. However, vaccinations in cancer patients require some further consideration [5].

Recently, the COVID-19 pandemic became a challenge for cancer patients and the oncological healthcare system worldwide. The pandemic affects the screening, diagnosis, follow up, and treatment schedule for cancer patients [6]. Moreover, COVID-19 affects physical and mental health in medical professionals and oncological patients [7,8,9]. The oncological treatment and diagnostic guidelines had to be reviewed and tailored to the new reality [10]. Since the beginning of the pandemic, the invention of the vaccine has been the main focus of research. In December 2020, the European Medicines Agency (EMA) authorized the first COVID-19 vaccine in Europe [11]. A newly developed mRNA vaccine conferred 95% protection against COVID-19 in general population. However, there is a lack of data concerning the safety and efficacy of the vaccine in cancer patients, who were excluded from clinical trials [11,12].

Patients with cancer are at high risk of severe COVID-19 complications. The probability of death among oncological patients with COVID-19 was estimated to be 25.6% [13]. Moreover, recent chemotherapy increases the risk of severe COVID-19 infection [14]. Analysis of the course of COVID-19 among patients with cancer supports giving them a high priority for vaccination, especially because seroconversion in patients with cancer infected by SARS-CoV-2 did not differ in comparison to the healthy population [15,16]. The European Society of Medical Oncology (ESMO) released statements concerning COVID-19 vaccination in oncological patients, which supports vaccination, the monitoring of side effects, and education in patients with cancer [17].

Vaccines and vaccination as a method for infectious disease prevention have always raised doubts, leading to anti-vaccination ideology and anti-vaccination movements. The reasons for refusing vaccination include negative attitudes towards vaccination and fear of side effects and needles [18,19]. Despite the consequences of the COVID-19 pandemic, there is a population of corona-sceptics that denies the existence of the disease and the need for vaccinations or preventive strategies, like wearing masks or social distancing [20,21]. The development of the new vaccines in a relatively short time, with the use of novel techniques, raised questions among the public. Concerns about the risk of side effects, safety and effectiveness are the main fears among cancer patients [22]. The usage of non-scientifically proven sources of information often leads patients to develop wrong perceptions and erroneous beliefs [23,24]. Data on the attitude of cancer patients towards SARS-CoV-2 vaccine is scarce.

In our study, we performed an online survey among Polish patients with a cancer diagnosis. The main aim of our study was to assess their acceptance of the vaccine and to analyze factors influencing patients’ attitudes. We aimed to analyze patients’ opinions and attitudes towards vaccinations in general, as well as towards vaccination against influenza and COVID-19.

## 2. Materials and Methods

### 2.1. Study Design and Participants

The data presented and compiled in this study were obtained on the initiative of the Young Oncologists Section of the Polish Society of Clinical Oncology through an anonymous online questionnaire addressed to patients with a cancer diagnosis. The participants were sought out through patients’ organizations, oncological clinics and social media of organizations involved in cancer. The patients were volunteers who agreed to fill out the questionnaire. All patients who were in the stages of before, during or after cancer treatment were eligible to participate. The survey was launched on 26 January 2021 and closed on 18 February 2021. During the study period, vaccinations against COVID-19 were being conducted in Poland only in healthcare workers and people over 70-years of age. The questionnaire was distributed by patients’ organizations, oncological clinics and promoted through social media and websites.

The questionnaire consisted of 39 anonymous open, semi-open, and closed questions with one correct answer or with a set of possible answers. The scope and subject matter of the survey questions were constructed in such a way as to preserve the anonymity of the respondent. The survey was developed based on past research involving attitudes and behaviors about vaccinations [25,26]. The study assessed the demographic characteristics of participants, including information on cancer diagnosis and treatment status, personal experiences with COVID-19, general attitudes towards vaccines and personal opinions on the COVID-19 vaccine. The translated version of a questionnaire is available as supplementary material (Supplementary materials—questionnaire).

The database was created as a result of the received questionnaires, which allowed for multi-level analysis of the collected material.

Opinions about vaccination in general and COVID-19 vaccination in particular were measured based on the assessment of specific statements on the Likert scale. Response options ranged from 1 (strongly disagree) to 5 (strongly agree). For reporting and analysis, responses 1 and 2 were grouped together as “disagree”, while 4 and 5 as “agree”. Response 3 was coded as “neither disagree nor agree”.

Patients’ attitudes towards vaccination against COVID-19 were analyzed based on the question “Do you want to get vaccinated against COVID 19?”. Responses “Definitely yes” and “Probably yes” were grouped together as “willing” while “Probably not” and “Definitely not” as “unwilling”.

### 2.2. Statistical Analysis

Discrete variables were summarized as numbers and percentages, continuous ones—with mean and standard deviation in the case of a normal distribution or with median and interquartile range when the distribution was skewed. Multivariable multinominal regression models were created to examine the association of socio-demographic, cancer-related, and COVID-19-related factors with general attitude toward vaccination and unwillingness or uncertainty about COVID-19 vaccination. In the first model, a “positive” attitude was coded as a reference category and compared with “negative” and “neutral” attitudes. In the second model, patients “willing” to vaccinate against COVID-19 were coded as a reference category and compared with “unwilling” and “undecided”. For multinominal regression models, a Pearson Chi-square test was used to evaluate the model’s goodness-of-fit, while Likelihood Ratio Chi-square tests were performed to evaluate the impact of each factor. Odds ratios (OR) with 95% Confidence Intervals (CI) were used to report the results of the regression model.

All analyses and figures were created using IBM SPSS Statistics for Windows version 26 (IBM Corp, Armonk, NY, USA). The differences were considered statistically significant if the *p* values were <0.05.

## 3. Results

### 3.1. Study Population

A total number of 635 respondents filled out the online questionnaire and were included in the analysis. Participants’ median age was 53 years (range 18–89 years) and 80.2% were women. Most of the respondents lived in a city with over 100.000 inhabitants (37.6%), while 22.2% lived in villages. The majority of participants had higher education (46.8%), were professionally active (46.5%), and in a relationship (71.2%).

The participants were diagnosed with cancer between 1982 and 2021, with 61.9% being actively treated at the survey time. The majority of respondents had a diagnosis of breast cancer (46.5%), followed by gastrointestinal (11.2%) and genitourinary cancer (8.8%).

More than half of the participants assessed their health as good or very good, compared to their peers, while over 60% of them had at least one comorbidity with cardiovascular disease as the most common one. One-hundred participants (15.7%) had COVID-19 infection before participating in the study, but only less than one percent required hospitalization. Of the patients, 49.9% and 15.4% knew someone suffering or dying of COVID-19 infection, respectively. As for COVID-19 related sanitary restrictions, more than 96% reported compliance with wearing a mask and washing and disinfecting hands regularly. Detailed socio-demographic characteristics of the study population are presented in Table 1.

### 3.2. General Attitudes and Opinions about Vaccinations

Only 10.1% of the responders (n = 64) have received regular seasonal vaccinations against influenza, 29.4% (n = 187) had been vaccinated, but not regularly, while the remaining 60.5% (n = 384) had never received the vaccine. Patients who have never been vaccinated against influenza were asked about the reasons for their attitudes. The most common reason was lack of information from physicians about the indication for flu vaccination (42.5%, 152/358), followed by the belief that that influenza is a mild seasonal illness that does not require vaccination (26.8%, 96/358) and low effectiveness (17.3%, 62/358). Other reasons were: serious complications after other vaccination (6.2%, 22/358), fear of the side effects (10.5%; 38/358), oncological contraindications (10.5%; 38/358), and general contraindications (2.2%; 8/358). Of the respondents 44.6% (n = 283) received other recommended vaccinations (e.g., against pneumococci or viral hepatitis).

To evaluate the overall opinion about vaccinations, study participants were asked to assess several statements on a Likert scale (Table 2). The majority of respondents thought that vaccinations are the most effective way to protect against serious infectious diseases. Around 70% of participants agreed on vaccinations being safe. Strikingly, only around half of the respondents declared being adequately informed about the vaccines’ side effects before vaccination. Around half of the participants disagreed that vaccines could potentially cause serious developmental disorders, for example, autism in children, while over 20% believed this. Of the respondents, 70.9% agreed that vaccinations have more advantages than disadvantages.

Overall, the majority of the study participants had a positive attitude towards vaccinations (73.7%, n = 468). Only 8.5% (n = 54) presented negative and 17.8% (n = 113) neutral opinions on this topic.

We have constructed a multivariable multinominal logistic regression model to identify factors predicting negative or neutral attitudes (model goodness-of-fit Pearson Chi-square *p =* 0.278, Cox and Snell pseudo-R square 0.275) (Table 3). Significant predictors in Likelihood Ratio tests associated with a negative or neutral opinion were education (*p =* 0.011), place of residence (*p =* 0.005), and obtaining information about vaccination from physicians (*p =* 0.001) or scientific guidelines (*p =* 0.002). Specifically, patients with primary education (OR 14.09, 95%CI 1.29–153.61) and who were unemployed (OR 3.51, 95%CI 1.06–11.62) were more likely to have a negative attitude towards vaccines. Patients respecting physicians’ authority in terms of vaccination information were less likely to present a negative (by 86%) or neutral (by 66%) rather than a positive attitude towards the vaccination.

### 3.3. Attitudes and Opinions about COVID-19 Vaccination

In the last part of the survey, we asked about the attitude towards vaccinations against COVD-19. We have found that 38.3% of the responders did not feel adequately informed about the COVID-19 vaccination. Moreover, around 40% of participants expressed concerns about vaccine side effects, effectiveness, composition, and rapid development (Table 4). Over 65% of responders agreed that patients with active oncological disease should be prioritized in vaccination programs, while 45.7% claimed that COVID-19 vaccination should be mandatory for patients with cancer (Table 4).

To assess patients’ needs for reliable information about COVID-19, we asked whether they have spoken with their general practitioners (GPs) or attending physicians (oncologist, radiologist or surgeon) about vaccinations against COVID-19. Of the participants, 26.8% (n = 170) and 43.6% (n = 277) spoke to their GP or attending physician, respectively. Only in less than five percent of the respondents had the doctor initiated the conversation. Of the respondents, 44.6% and 40.8% (n = 283; n = 259), had not spoken to their GP or treating physician, respectively, about vaccination against COVID-19 but would like to. In participants who had such a conversation, recommendations to get vaccinated were given by 65.3% (111/170) of GPs and 79.1% (219/277) of oncologists.

**Figure 1:**
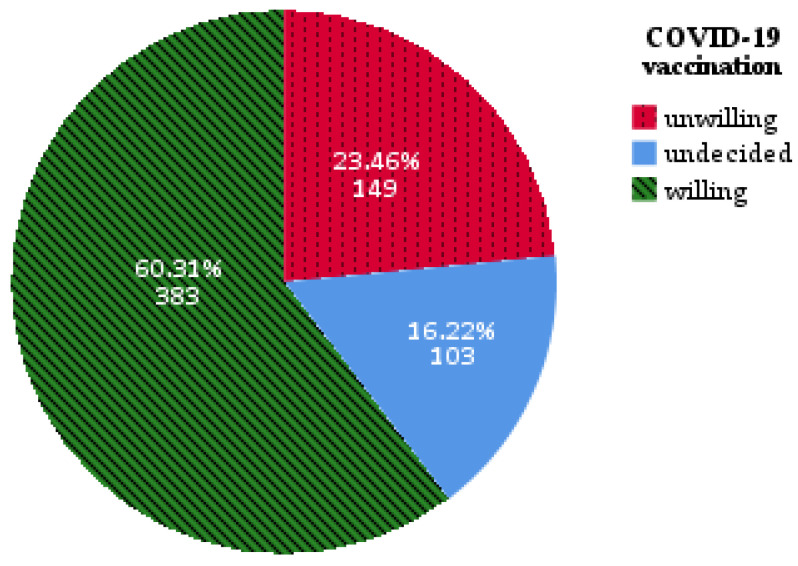
Patients’ willingness to get vaccinated against COVID-19.

At the time of the study, 6.8% (n = 43) of participants were already vaccinated against COVID-19, and 60.31% (n = 383) declared the will to get vaccinated. Of the respondents, 23.46% (n = 149) were unwilling to get vaccinated and 16.22% (n = 103) were undecided (Figure 1).

To better understand the opinions of patients with objections to the SARS-CoV-2 vaccine, we decided to take a closer look at their answers about their opinions on vaccinations (Supplementary Tables S1 and S2). The majority of the respondents in this population were afraid of the COVID-19 vaccine’s side effects (66.4%) and the short time of development (79.9%). Interestingly, patients who were skeptical about the vaccine did not feel sufficiently informed about its side effects and medical indications in cancer patients (64.4%).

**Figure 2:**
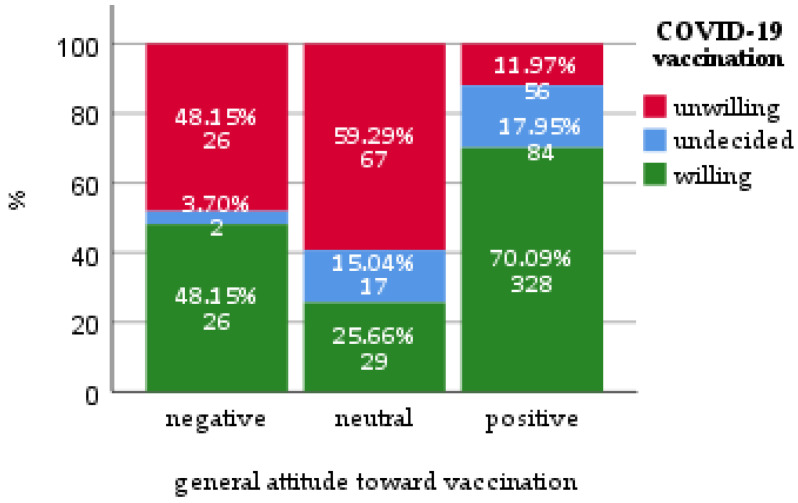
Willingness to get vaccinated against COVID-19 stratified by a general attitude toward vaccination.

We have constructed a multivariable multinominal logistic regression model to identify factors predicting negative or neutral attitude (model goodness-of-fit Pearson Chi-square *p =* 0.443, Cox and Snell pseudo-R square 0.388) (Table 5). Significant predictors in Likelihood Ratio tests were education (*p =* 0.02), marital status (*p =* 0.014), active anticancer treatment (*p =* 0.009), vaccination against influenza (*p =* 0.006) and general attitude towards vaccination (*p* < 0.001). Specifically, patients with negative (OR 3.25, 95%CI 1.41–7.50) or neutral (OR 13.34, 95%CI 6.80–26.17) attitudes towards vaccinations, in general, tended to have rather a negative attitude towards COVID-19 vaccination (Figure 2). Additionally, patients currently undergoing anti-cancer treatment had lower odds of being undecided about the SARS-CoV-2 vaccine (OR 0.38, 95%CI 0.21–0.71).

## 4. Discussion

Here, we present the results of one of the few studies addressing the opinions, expectations, and fears of cancer patients regarding the COVID-19 vaccine [22,27]. By understanding the patients’ perceptions of the vaccine, physicians and care providers can better address patients’ needs and promote and encourage COVID-19 vaccinations. Even before the SARS-CoV-2 pandemic, there were several obstacles to successful vaccination programs among cancer patients [28]. Despite clear recommendations to vaccinate patients receiving anti-cancer chemotherapy against preventable infections, for example, influenza, cancer patients’ vaccination rates remained low [29,30,31].

Our study shows that the majority of cancer patients are willing to get vaccinated against COVID-19, which stays in line with surveys conducted by Barriere et al. [27] and Kelkar et al [22]. Moreover, most of the patients actively seek information on the vaccine and value their physicians’ opinion in this area. This finding has been also confirmed by both the abovementioned studies [22,27], which underlines the role of clinical oncologists in encouraging vaccine acceptance among patients.

The importance of clinicians’ support in patients’ decision was also reported for other vaccines such as influenza [32,33]. Data from studies on influenza show that recommendation from a patient’s provider results in a 7-fold greater likelihood of vaccination [31]. On the other hand, our survey revealed the harsh reality of the COVID-19 pandemic, where clinical oncologists struggling with limited time and resources often fail to meet patients’ needs for information about the COVID-19 vaccine. Only half of the respondents felt adequately informed about the side effects of the vaccine. Approximately 40% of the patients in our survey spoke to their attending physician about the vaccination, but only in less than five percent of the cases had the doctor initiated the conversation. This, however, can partially be explained by the fact that, at the time of the survey, oncological patients in Poland were not yet included in the national vaccination program.

Interestingly, the vast majority of respondents agreed that cancer patients should be prioritized in vaccination efforts, which is an opinion that is shared by oncological associations including ESMO, the American Society of Clinical Oncology (ASCO) and the Association of American Cancer Institutes (AACI) [12,15,17].

A strong predictor of COVID-19 vaccine acceptance was previous influenza vaccination and respect for a physician’s authority, which was also shown in Barriere et al. study. The latter underlines once more the crucial role of the physician in patient education and forming attitudes towards vaccination. A history of influenza vaccination seems to be a reliable predictor of COVID-19 vaccine acceptance, which was confirmed in cancer patients [27] as well as in the general population [26,34].

Our survey also revealed the main fears presented by cancer patients with negative attitudes towards the vaccine, which included fear of the side effects, the short time of development and insufficient information. Previous surveys dedicated to the influenza vaccine among cancer patients revealed almost identical stances [35]. What is noteworthy is the fact that all the above mentioned fears can be addressed by educational measures with good outcomes. This was recently documented in a study assessing the impact of a SARS-CoV-2 webinar on cancer patients [22]. Because of the influence of previous influenza vaccines on the acceptance of the COVID-19 vaccine, one can hypothesize that these educational actions can contribute to long-term attitudes to vaccinations and result in higher vaccination rates for other infectious diseases.

When interpreting the results of this study, a few limitations should be taken into account. The majority of the patients were female, with higher education, living in large cities and who were professionally active. Overrepresentation of these populations was most likely influenced by the fact that the survey was carried out via the Internet. Patients who do not have the ability to use the Internet could not therefore be reached by this survey. However, considering the restrictions imposed by the COVID-19 pandemic, we decided to carry out the survey online. Thus, we were not able to recruit patients to the study in a structured way, which imposed a possible selection bias that needs to be considered when analyzing the data. The ongoing vaccination program organized by the Polish Ministry of Health could also influence patients’ attitudes towards the SARS-CoV-2 vaccine.

## 5. Conclusions

Overall, in our study, we observed high rates of positive attitudes towards vaccinations and a high rate of willingness to be vaccinated against COVID-19. On the other hand, vaccination rates against influenza remain low. Notably, a high proportion of patients feel not adequately informed about several aspects of vaccines, starting from research and development to their indications, efficacy, and side effects. This is a crucial factor leading to vaccine hesitancy.

Considering that, nowadays, COVID-19 and cancer are the main threats to human health, increased efforts should be put into patient education about vaccination. Several parties, including physicians of all specialties, nurses, patients’ organizations, stakeholders and media, should be engaged. It should be underlined that COVID-19 vaccination in cancer patients not only protects from infection or severe complications but can also allow patients to continue and complete oncological treatment as planned without unnecessary interruption, which leads to better long-term outcomes.

Lastly, we would like to highlight that a generally positive attitude towards vaccination predicts a higher acceptance rate of the COVID-19 vaccine. This observation can have potential long-term implications for the future if a new vaccine against another potentially threatening disease becomes available.

## Figures and Tables

**Table 1:** Socio-demographic characteristics of the study population.

Characteristic	Parameter	Study Population n (%), n = 635
Age	Median years (range)	53 (18–89)
Gender	Male	124 (19.5)
	Female	509 (80.2)
	Do not want to provide information	2 (0.3)
Place of residence	Village	141 (22.2)
	City < 50,000 inhabitants	182 (28.7)
	City 50,000–100,000 inhabitants	73 (11.5)
	City > 100,000 inhabitants	239 (37.6)
Education	Primary	13(2.0)
	Vocational	65 (10.2)
	Secondary	253(39.8)
	Higher	296 (46.8)
	Do not want to provide information	8 (1.3)
Occupational situation	Professionally active	295 (46.5)
	Retired	181 (28.5)
	On a disability pension	77 (12.1)
	Unemployed	54 (8.5)
	Student	15 (2.4)
	Do not want to provide information	13 (2.0)
Marital status	In a relationship	452 (71.2)
	Single	61 (9.6)
	Divorced	58 (9.1)
	Widow/widower	58 (7.1)
	Do not want to provide information	16 (2.5)
Cancer type	Breast cancer	295 (46.5)
	Gastrointestinal cancers	71 (11.2)
	Urogenital cancers	56 (8.8)
	Hematological	51 (8.0)
	Gynecological	49 (7.7)
	Sarcoma	38 (6.0)
	Thoracic tumors	38 (6.0)
	Head and neck cancers	18 (2.8)
	Melanoma	11 (1.7)
	Other	8 (1.3)
Active anti-cancer treatment		393 (61.9)
How do you evaluate your health in comparison to your peers?	Very good	73 (11.5)
	Good	264 (41.6)
	Medium	227 (35.7)
	Bad	63 (9.9)
	Very bad	8 (1.3)
Comorbidities	Overall	388 (61.1)
Comobidities	Cardiovascular diseases	233 (36.7)
	Respiratory tract disease	61 (9.6)
	Autoimmune diseases	86 (13.5)
	Neurological diseases	28 (4.4),
Allergies		171 (26.9)
History of COVID-19 infection		100 (15.7)
COVID-19 hospitalization		5 (0.9%)
Know someone who suffered from COVID-19		317 (49.9)
Know someone who died of COVID-19		98 (15.4)
Wearing mask or face shield		610 (96.1)
Pandemic recommendations compliance (washing hands etc.)		612 (96.4)
The main source of information about the world:	Websites	281 (44.3)
	TV and radio	230 (36.2)
	Social media	54 (8.5)
	Professional literature	47 (7.4)
	Press	14 (2.2)
	Friends and family	9 (1.4)
Source of information about vaccinations (multiple choice):	TV and radio	316 (49.8)
	Websites	286 (45.0)
	Social media	134 (21.1)
	Physician	122 (20.9)
	Scientific Guidelines	125 (19.7)
	Professional literature	109 (17.2)
	Friends/family	78 (12.3)
	Press	63 (9.9)
	Patient’s organizations articles	58 (9.1)
TV use	I do not watch TV	100 (15.7)
	Less than one hour	99 (15.6)
	Between 1 and 2 h	195 (30.7)
	Between 2 and 4 h	167 (26.3)
	More than 4 h	74 (11.7)
Internet use	I do not use the Internet	51 (8.0)
	Less than one hour	87 (13.7)
	Between 1 and 2 h	262 (41.3)
	Between 2 and 4 h	175 (27.6)
	More than 4 h	60 (9.4)
Social media use	Yes, everyday	432 (68.0)
	Yes, several times a week	88 (13.9)
	Yes, but less than once a week	19 (3.0)
	No	96 (15.1)

**Table 2:** Cancer patients’ general opinions on vaccinations.

Question	Answer n (%), n = 635		
	Disagree (Likert Scale 1–2)	Neither Disagree Nor Agree (Likert Scale 3)	Agree (Likert Scale 4–5)
Thanks to preventive vaccinations many dangerous diseases are practically non-existent today.	43 (6.8)	59 (9.3)	533 (83.9)
Vaccinations are the most effective way to protect against serious infectious diseases.	48 (7.6)	59 (9.3)	528 (83.1)
Vaccinations are safe.	66 (10.4)	117 (18.4)	452 (71.2)
Before vaccination, patients are adequately informed about the side effects.	171 (26.9)	162 (25.5)	302 (47.6)
Vaccinations are promoted not because they are really needed but because it is in the interests of pharmaceutical companies.	334 (52.6)	147 (23.1)	154 (24.3)
Vaccinations in children can cause serious developmental disorders, e.g., autism.	368 (58.0)	134 (21.1)	133 (20.9)
Vaccinations have more advantages than disadvantages.	76 (12.0)	109 (17.2)	450 (70.9)

**Table 3:** Predictors of negative and neutral attitude towards vaccination using multivariable multinominal logistic regression. Significant factors are in bold.

Factor	Negative Attitude towards Vaccination		Neutral Attitude towards Vaccination	
	OR	95% CI	OR	95% CI
Female (ref. male)	3.69	0.83–16.33	2.24	0.92–5.43
Age (ref. ≤44)				
45–64	0.93	0.32–2.73	0.74	0.38–1.44
65+	0.17	0.03–1.12	0.42	0.11–1.61
Education (ref. secondary)				
primary	**14.09**	**1.29–153.61**	3.20	0.68–15.06
basic vocational	1.97	0.55–7.11	1.17	0.47–2.91
higher	**0.26**	**0.10–0.68**	0.78	0.43–1.40
Place of living (ref. village)				
city < 50,000 inhabitants	1.91	0.65–5.61	0.88	0.45–1.71
city 50,000–100,000 inhabitants	0.71	0.14–3.71	0.68	0.28–1.61
city > 100,000 inhabitants	1.19	0.39–3.64	**0.29**	**0.14–0.59**
Marital status (re. single)				
in a relationship	2.48	0.67–10.77	1.08	0.49–2.35
widow/widower	3.27	0.42–25.62	2.33	0.67–8.16
divorced	4.48	0.74–27.28	1.93	0.63–5.85
Occupational status (ref. professionally active)				
retired	2.30	0.65–8.01	0.53	0.19–1.53
on a disability pension	1.22	0.34–4.36	1.07	0.47–2.45
unemployed	**3.51**	**1.06–11.62**	2.21	0.93–5.21
studying	0.57	0.4–8.2	2.67	0.66–10.80
Main source of information (ref. radio and TV)				
Internet services	1.95	0.65–5.90	1.21	0.59–2.47
Social media	1.36	0.30–6.18	1.25	0.43–3.63
Friends and family	4.84	0.22–107.29	1.12	0.11–11.20
Specialistic literature	**16.00**	**2.77–92.44**	**3.60**	**1.11–11.63**
Time spent watching TV (ref. 1–2 h)				
I do not watch TV	1.13	0.34–3.78	1.85	0.83–4.12
Less than one hour	0.55	0.17–1.81	0.75	0.35–1.61
Between 2 and 4 h	0.57	0.19–1.72	0.51	0.25–1.03
More than 4 h	0.45	0.1–2.06	**0.33**	**0.12–0.96**
Time spent surfing the Internet (ref. 1–2 h)				
I do not watch TV	4.60	0.34–62.02	1.26	0.33–4.75
Less than one hour	1.02	0.28–3.75	0.92	0.43–2.00
Between 2 and 4 h	0.62	0.24–1.61	0.59	0.31–1.15
More than 4 h	0.97	0.24–3.95	1.30	0.51–3.30
Use of social media (ref. few times a week)				
everyday	1.04	2.95–3.69	0.68	0.32–1.43
less than once a week	0.38	0.02–6.31	1.10	0.24–5.04
no	0.25	0.02–3.32	1.00	0.33–3.05
Use of articles from patients’ organizations as a source of information about vaccinations (ref. No)	1.17	0.26–5.16	0.67	0.24–1.88
Use of radio and TV as a source of information about vaccinations (ref. No)	0.68	0.25–1.85	0.95	0.48–1.89
Use of Internet about vaccinations (ref. No)	1.20	0.52–2.78	0.68	0.38–1.20
Physician as a source of information about vaccinations (ref. No)	**0.14**	**0.03–0.61**	**0.34**	**0.16–0.74**
Scientific Literature as a source of information about vaccinations (ref. No)	0.97	0.31–3.06	0.43	0.19–1.01
Guidelines as a source of information about vaccinations (ref. No)	**0.14**	**0.03–0.60**	**0.4**	**0.19–0.86**
Friends and family as a source of information about vaccinations (ref. No)	1.00	0.23–4.26	2.27	1.08–4.80
Social media as a source of information about vaccinations (ref. No)	2.60	1.02–6.64	0.89	0.44–1.78
Press as a source of information about vaccinations (ref. No)	0.66	0.10–4.24	1.02	0.39–2.66
General assessment of personal health (ref. very good)				
Good	1.12	0.33–3.81	0.98	0.43–2.22
Medium	0.76	0.21–2.80	0.74	0.31–1.75
Bad	1.46	0.31–6.90	0.49	0.15–1.56
Very bad	1.53	0.10–23.8	0.76	0.06–9.00
Concomitant diseases (ref. No)	0.65	0.28–1.52	0.95	0.55–1.64
Active anticancer treatment (ref. No)	1.27	0.54–2.99	1.10	0.62–1.95
Cancer type (Ref. gastrointestinal)				
Melanoma and skin cancer	0.96	0.08–11.62	1.44	0.21–9.70
Sarcoma	2.90	0.44–19.10	1.84	0.50–6.75
Gynecological	0.95	0.16–5.59	0.70	0.19–2.53
Head and neck	NA	NA	0.39	0.06–2.45
Hematological	1.17	0.17–8.23	0.70	0.18–2.69
Genitourinary	0.53	0.06–4.43	1.18	0.39–3.60
Respiratory tract	1.24	0.20–7.48	0.76	0.19–3.01
Breast	0.36	0.08–1.64	0.68	0.25–1.86
Other	1.72	0.05–64.21	1.27	0.09–17.74

**Table 4:** Attitudes of patients with cancer towards COVID-19 vaccination.

Question	Answer *n* (%), n = 635		
	Disagree (Likert Scale 1–2)	Neither Disagree Nor Agree (Likert Scale 3)	Agree (Likert Scale 4–5)
I am afraid of the vaccine’s side effects.	198 (31.2)	153 (24.1)	284 (44.7)
I have concerns about the effectiveness of the vaccine.	235 (37.0)	118 (18.6)	282 (44.4)
I am afraid of the composition of the vaccine.	284 (44.7)	112 (17.6)	239 (37.6)
The vaccine was developed too rapidly.	239 (37.6)	102 (16.1)	294 (46.3)
The vaccine contains bodies of aborted children.	502 (79.1)	84 (13.2)	49 (7.7)
Religious reasons prevent me from vaccination.	592 (93.2)	23 (3.6)	20 (3.1)
Coronavirus does not exist, so I do not need to get vaccinated.	541 (85.2)	52 (8.2)	42 (6.6)
I do not need to get vaccinated because I believe that the risk of getting sick in my case is low because I adhere to the recommendations of isolation and have no contact with other people.	470 (74.0)	92 (14.5)	73 (11.5)
I believe that patients in active cancer treatment should get vaccinated first.	109 (17.2)	101 (15.9)	425 (66.9)
I believe that COVID-19 vaccination should be mandatory.	232 (36.5)	113 (17.8)	290 (45.7)
I am sufficiently informed about the possibilities and safety of the vaccination in cancer patients.	243 (38.3)	130 (20.5)	262 (41.3)
I believe that cancer patients should not get vaccinated against COVID-19	419 (66.0)	122 (19.2)	94 (14.8)

**Table 5:** Predictors of uncertainty and unwillingness to vaccinate against COVID-19 using multivariable multinominal logistic regression. Significant factors are in bold.

Factor	Unwilling to Vaccinate against COVID-19		Undecided to Vaccinate against COVID-19	
	OR	95% CI	OR	95% CI
Female (ref. male)	**2.62**	**1.01–6.80**	0.93	0.39–2.22
Age (ref. ≤44)				
45–64	0.58	0.28–1.18	0.54	0.25–1.15
65+	**0.22**	**0.06–0.82**	0.27	0.07–1.04
Education (ref. secondary)				
primary	0.76	0.08–7.29	1.72	0.37–7.97
basic vocational	1.32	0.55–3.15	0.33	0.11–1.01
higher	**0.45**	**0.24–0.83**	0.75	0.40–1.41
Place of living (ref. village)				
city < 50,000 inhabitants	2.01	0.96–4.20	0.70	0.33–1.51
city 50,000–100,000 inhabitants	0.69	0.25–1.95	0.84	0.34–2.06
city > 100,000 inhabitants	1.16	0.54–2.47	0.83	0.41–1.71
Marital status (re. single)				
in a relationship	0.44	0.19–1.00	0.51	0.21–1.26
widow/widower	**0.23**	**0.06–0.89**	0.40	0.10–1.57
divorced	**0.20**	**0.06–0.66**	**0.11**	**0.02–0.48**
Occupational status (ref. professionally active)				
retired	1.39	0.54–3.57	0.95	0.35–2.59
on a disability pension	0.75	0.31–1.79	0.82	0.34–1.97
Unemployed	0.77	0.31–1.94	0.38	0.12–1.18
studying	0.40	0.07–2.21	NA	NA
Main source of information (ref. radio and TV)				
Internet services	0.95	0.45–2.00	0.73	0.35–1.54
social media	0.52	0.18–1.51	0.57	0.18–1.84
Friends and family	0.70	0.06–8.50	0.44	0.03–5.78
Specialistic literature	2.45	0.76–8.16	0.95	0.26–3.46
Press	0.76	0.10–5.96	1.29	0.25–6.52
Time spent watching TV (ref. 1–2 h)				
I do not watch TV	1.28	0.53–3.08	**0.36**	**0.13–0.99**
Less than one hour	2.13	0.92–4.94	0.78	0.34–1.82
Between 2 and 4 h	**2.54**	**1.19–5.42**	0.80	0.40–1.60
More than 4 h	1.73	0.61–4.90	0.88	0.35–2.21
Time spent surfing the Internet (ref. 1–2 h)				
I do not watch TV	0.40	0.08–1.84	1.72	0.47–2.27
Less than one hour	0.66	0.27–1.60	1.08	0.45–2.61
Between 2 and 4 h	0.81	0.41–1.56	1.02	0.53–1.97
More than 4 h	0.73	0.28–1.87	0.78	0.26–2.35
Use of social media (ref. few times a week)				
everyday	1.06	0.46–2.44	1.69	0.71–4.01
less than once a week	0.89	0.16–4.90	0.52	0.08–3.42
No	0.89	0.26–3.10	1.04	0.32–3.35
Use of articles from patients’ organizations as a source of information about vaccinations (ref. No)	0.85	0.31–2.36	0.66	0.29–2.17
Use of radio and TV as a source of information about vaccinations (ref. No)	0.74	0.36–1.50	0.92	0.45–1.88
Use of Internet about vaccinations (ref. No)	1.19	0.66–2.14	1.05	0.57–1.94
Physician as a source of information about vaccinations (ref. No)	0.80	0.37–1.71	0.98	0.49–1.96
Scientific Literature as a source of information about vaccinations (ref. No)	0.51	0.22–1.18	0.55	0.23–1.32
Guidelines as a source of information about vaccinations (ref. No)	0.91	0.68–3.53	1.00	0.46–2.17
Friends and family as a source of information about vaccinations (ref. No)	1.55	0.68–3.53	1.90	0.85–4.25
Social media as a source of information about vaccinations (ref. No)	1.42	0.72–2.80	1.06	0.52–2.19
Press as a source of information about vaccinations (ref. No)	0.99	0.36–2.77	0.61	0.24–1.57
General assessment of personal health (ref. very good)				
Good	0.66	0.28–1.55	1.59	0.60–4.20
Medium	1.25	0.52–3.02	1.83	0.67–5.00
Bad	0.80	0.25–2.54	**3.54**	**1.08–11.55**
Very bad	1.92	0.24–15.57	NA	NA
Concomitant diseases (ref. No)	1.10	0.62–1.97	1.14	0.65–2.00
Active anticancer treatment (ref. No)	0.83	0.45–1.53	**0.38**	**0.21–0.71**
Cancer type (Ref. gastrointestinal)				
Melanoma and skin cancer	0.67	0.08–5.52	1.91	0.23–16.24
Sarcoma	1.06	0.26–4.27	0.59	0.12–2.83
Gynecological	0.34	0.10–1.18	0.50	0.12–2.05
Head and neck	0.42	0.06–3.17	1.30	0.27–6.32
Hematological	0.36	0.09–1.42	0.55	0.13–2.22
Genitourinary	0.66	0.19–2.32	0.98	0.32–2.97
Respiratory tract	1.15	0.30–3.38	2.47	0.76–8.04
Breast	0.45	0.17–1.20	0.67	0.24–1.86
Other	NA	NA	0.26	0.02–4.46
Had COVID-19 infection (Ref. No)	2.03	0.99–4.11	0.83	0.36–1.9–
Know somebody who had COVID-19 infection (Ref. No)	0.69	0.37–1.32	**0.50**	**0.26–0.96**
Know somebody who died of COVID-19 (Ref. No)	1.53	0.69–3.40	1.30	0.57–2.98
Vaccination against influenza (Ref. never)				
regularly every year	**0.28**	**0.09–0.91**	0.46	0.18–1.17
not regularly	**0.36**	**0.19–0.70**	0.61	0.33–1.11
General attitude towards vaccination (Ref. positive)				
Negative	**3.25**	**1.41–7.50**	0.28	0.06–1.38
Neutral	**13.34**	**6.80–26.17**	1.99	0.90–4.39

## Data Availability

Data will be available from the corresponding author upon reasonable request.

## Supplementary Materials

The following are available online at https://www.mdpi.com/article/10.3390/vaccines9050411/s1, Questionnaire, Supplementary Table S1. Opinions about vaccinations in general in the subgroup of patients unwilling to get vaccinated against COVID-19 (n = 125); Supplementary Table S2. Opinions about vaccinations in general in the subgroup of patients unwilling to get vaccinated against COVID-19 (n = 125).
