# Supplementary material for: Attitudes of Patients with Cancer towards Vaccinations—Results of Online Survey with Special Focus on the Vaccination against COVID-19
Source: Vaccines (Basel). 2021 Apr 21;9(5):411. doi: 10.3390/vaccines9050411 (PMC8142983; doi:10.3390/vaccines9050411)
Supplement: Supplementary file 1 [file vaccines-09-00411-s001.zip › Supplementary materials 1 ¿C questionnaire.pdf]

Supplementary materials – The translation of online survey questionnaire

Dear Sir/Madam,

We would like to ask you to complete a questionnaire regarding the opinion of cancer patients on vaccinations, including vaccines against COVID-19.
We are young doctors belonging to the Section of Young Oncologists of the Polish Society of Clinical Oncology. In our daily work, we treat patients suffering from malignant tumors. Our patients often ask us for opinions on vaccination against the coronavirus. We have created a survey that will provide a better understanding of patients' attitudes to immunization, enabling us to develop tailored educational materials.
The survey is aimed at patients with cancer who are currently undergoing treatment and those who have already completed cancer treatment and are under observation.
It should take about 10-15 minutes to complete the survey. The survey is completely anonymous.
We will be grateful for completing the questionnaire and passing it on to other patients.

General information

1. What is your age:…………..

2. What is your gender:
a) Male
b) Female
c) I don't want to give information about my gender

3. What is your place of residence:
a) village
b) city with up to 50,000 inhabitants
c) city with 50,000- 100,000 inhabitants
d) city with over 100,000 inhabitants

4. What is your marital status:
a) Single
b) In a relationship
c) Divorced
d) Widow/widower
e) I do not want to provide information about my marital status

5. What is your level of education:
a) Primary education
b) Vocational school/trade school
c) Secondary education
d) Higher education
e) I do not want to provide information about my education

6. What is your professional situation?
a) Professionally inactive/unemployed
b) Professionally active
c) On a disability pension
d) Retired
e) Student
f) I do not want to provide information about my professional situation

7. What is your main source of information about the world?
a) Television and radio

b) Press
c) Internet websites
d) Social media
e) Friends and family
d) Professional literature

8. What is your main source of information on vaccination? (multiple choice)
a) Television and radio
b) Press
c) Internet websites
d) Social media
e) Friends and family
f) Professional literature
g) Physician
h) Articles of patient organizations
i) Guidelines of scientific societies
j) I am not interested in vaccinations

9. How much time do you spend watching TV every day?
a) I do not watch TV
b) Less than one hour
c) Between 1 and 2 hours
d) Between 2 and 4 hours
e) More than 4 hours

10. How many hours a day do you spend surfing the internet?
a) I'm not using the internet
b) Less than one hour
c) Between 1 and 2 hours
d) Between 2 and 4 hours
e) More than 4 hours

11. Do you use social media (e.g. Facebook, Twitter, Instagram)?
a) Yes, everyday
b) Yes, several times a week
c) Yes, but less than once a week
d) No

Cancer-related information

12. What is your primary cancer type?
a) Head and neck cancer (e.g. cancer of the larynx, throat)
b) A cancer of the respiratory system (e.g. lung cancer, pleural mesothelioma)
c) Gastrointestinal cancer (e.g. colorectal, stomach, liver, pancreatic cancer)
d) Breast cancer
e) Genitourinary cancer (e.g. cancer of the kidney, bladder, prostate, testes)
f) Gynecological cancer (e.g. uterine cancer, ovarian cancer)
g) Hematological cancer (e.g. leukemia, lymphoma, myeloma)
h) A tumor of the nervous system (e.g. glioma, meningioma)
i) Sarcoma
j) Melanoma or other skin cancer
k) Other, please specify:…………………………

13. Please enter the year of diagnosis of your cancer:…………………….

14. Are you currently receiving oncological treatment? (e.g. intravenous infusions, tablets, hormone therapy):
a) Yes
b) No

15. Are you currently in observation after completion of treatment?
a) Yes
b) No

General health

16. How do you evaluate your health in comparison to your peers?
a) Very good
b) good
c) Average
d) Bad
e) Very bad

17. Do you suffer from any comorbidities?
a) Yes
b) No

18. Do you suffer from cardiovascular diseases? (e.g. hypertension, past myocardial infarction, heart failure, atrial fibrillation)?
a) Yes
b) No

19. Do you suffer from respiratory diseases (e.g. asthma, COPD)?
a) Yes
b) No

20. Do you suffer from autoimmune diseases (e.g. Hashimoto's disease, lupus, inflammatory bowel disease)?
a) Yes
b) No

21. Do you suffer from neurological diseases (e.g. stroke, multiple sclerosis, myasthenia gravis)?
a) Yes
b) No

22. Do you have any allergies (e.g. food, drugs, insect venom, inhalants, pollen allergy)?
a) Yes
b) No

COVID-19-related information

23. Have you ever had COVID-19 infection?
a) Yes
b) No

24. Have you been hospitalized for COVID-19 infection?
a) Yes

b) No
c) I did not have COVID-19 infection

25. Do you know anybody in your closest vicinity who suffered from COVID-19 infection?
a) Yes
b) No

26. Do you know anybody in your closest vicinity who died from COVID-19?
a) Yes
b) No

27. Do you wear a mask and/or a face shield?
a) Yes
b) No

28. Do you follow the pandemic recommendations (e.g. washing and disinfecting hands, avoiding contact, keeping social distancing)?
a) Yes
b) No

General attitude towards vaccinations

29. Please rate your attitude to individual statements on a scale from 1 to 5 (1- strongly disagree, 5 - strongly agree)
a) Thanks to preventive vaccinations, many dangerous diseases are practically non-existent today.
b) Vaccinations are the most effective way to protect against serious infectious diseases.
c) Vaccinations are safe.
d) Prior to vaccination, patients are adequately informed about the side effects of the vaccines.
e) Vaccinations are promoted not because they are really needed, but because it is in the interests of pharmaceutical companies.
f) Vaccines for children can cause serious developmental disorders in them, e.g. autism.
g) Vaccinations have more advantages than disadvantages

30. How do you rate your overall attitude towards vaccination?
a) Very positive
b) Positive
c) Neutral
d) Negative
e) Very negative

31. Do you get vaccine against influenza ?
a) I get vaccinated regularly every year
b) I get vaccinated, but not regularly
c) I have never got vaccinated

32a. Why haven't you got vaccinated against influenza? (available to those patients who answered „c)" in the question 32; multiple answers can be selected)
a) Influenza is a mild seasonal illness that does not require vaccination
b) I got vaccinated once and I had serious complications, since then I do not get vaccinated
c) I am afraid of the side effects
d) I have not received information from my doctor about the indication for vaccination
e) the vaccine is not very effective (it does not protect against flu)
f) I have contraindications for vaccination

g) Due to my cancer, I was not vaccinated against influenza

32. Did you get vaccinated with any other recommended vaccine (e.g. against pneumococci, viral hepatitis)?
a) Yes
b) No

Attitude towards vaccination against COVID-19

33. Please rate your attitude to individual statements on a scale from 1 to 5 (1- strongly disagree, 5 - strongly agree)
a) I am afraid of complications after vaccination.
b) I have concerns about the effectiveness of the vaccine.
c) I am afraid of the composition of the vaccine.
d) The vaccine was developed too rapidly.
e) The vaccine contains the bodies of aborted children.
f) Religious reasons prevent me from vaccinating.
g) Coronavirus does not exist, so I don't need to get vaccinated.
h) I do not need to vaccinate because I believe that the risk of getting sick in my case is low, because I adhere to the recommendations of isolation and have no contact with other people.
i) I believe that patients with active cancer should be vaccinated first.
j) I believe that COVID-19 vaccination should be mandatory.
k) I am sufficiently informed about the possibilities and safety of vaccination in cancer patients.
l) I believe that cancer patients should not be vaccinated against COVID-19.

34. Are you already vaccinated against COVID19 at the time of completing the survey?
a) Yes
b) No

35. Do you want to get vaccinated against COVID 19?
a) Definitely yes
b) Probably yes
c) I do not know
d) Probably not
e) Definitely not

36. Have you spoken to your general practitioner about vaccination against COVID-19?
a) Yes, the doctor initiated a conversation
b) Yes, I asked the doctor about the vaccination
c) No, I don't need that
d) no, but I would like to

37. Has your general practitioner recommended you to be vaccinated against COVID-19?
a) Yes
b) No
c) I have not spoken to the doctor about COVID19 vaccination

38. Have you talked to your treating physician (oncologist, hematologist, surgeon, radiotherapist) about vaccination against COVID-19?
a) Yes, the doctor initiated a conversation
b) Yes, I asked the doctor about the vaccination
c) No, I don't need that
d) No, but I would like to

39. Has your attending physician (oncologist, hematologist, surgeon, radiotherapist) recommended you to be vaccinated against COVID-19?
a) Yes
b) No
c) I have not spoken to the doctor about vaccinations
