# Supplementary material for: Attitudes of Patients with Cancer towards Vaccinations—Results of Online Survey with Special Focus on the Vaccination against COVID-19
Source: Vaccines (Basel). 2021 Apr 21;9(5):411. doi: 10.3390/vaccines9050411 (PMC8142983; doi:10.3390/vaccines9050411)
Supplement: Supplementary file 1 [file vaccines-09-00411-s001.zip › Supplementary materials 2 - tables.pdf]

Supplementary Table S1. Opinions about vaccinations in general in the subgroup of patients unwilling to get vaccinated against COVID-19 (n=149).

	Disagree (Likert scale 1-2)	Neither disagree nor agree (Likert scale 3)	Agree (Likert scale 4-5)
Thanks to preventive vaccinations, many dangerous diseases are practically non-existent today.	13 (8.7)	31 (20.8)	105 (70.5)
Vaccinations are the most effective way to protect against serious infectious diseases.	19 (12.8)	34 (22.8)	96 (64.4)
Vaccinations are safe.	28 (18.8)	62 (41.6)	59 (39.6)
Prior to vaccination, patients are adequately informed about the side effects.	72 (48.3)	47 (31.5)	30 (20.1)
Vaccinations are promoted not because they are really needed, but because it is in the interests of pharmaceutical companies.	32 (21.5)	63 (42.3)	54 (36.2)
Vaccinations in children can cause serious developmental disorders, e.g. autism.	54 (36.2)	48 (32.2)	47 (31.5)
Vaccinations have more advantages than disadvantages	21 (14.1)	66 (44.3)	62 (41.6)

Supplementary Table S2. Opinions about vaccinations in general in the subgroup of patients unwilling to get vaccinated against COVID-19 (n=149).

	Disagree (Likert scale 1-2)	Neither disagree nor agree (Likert scale 3)	Agree (Likert scale 4-5)
I am afraid of the vaccine side effects	19 (12.8)	31 (20.8)	99 (66.4)
I have concerns about the effectiveness of the vaccine	14 (9.4)	30 (20.1)	105 (70.5)
I am afraid of the composition of the vaccine.	23 (15.4)	34 (22.8)	92 (61.7)
The vaccine was developed too quickly	12 (8.1)	18 (12.1)	119 (79.9)
The vaccine contains bodies of aborted children	98 (65.8)	36 (24.2)	15 (10.1)
Religious reasons prevent me from vaccinating	135 (90.6)	11 (7.4)	3 (2.0)
Coronavirus does not exist, so you don't need to get vaccinated	109 (73.2)	25 (16.8)	15 (10.1)
I do not need to vaccinate because I believe that the risk of getting sick in my case is low, because I adhere to the recommendations of isolation and have no contact with other people	63 (42.3)	55 (36.9)	31 (20.8)
I believe that patients in active cancer treatment should get vaccinated first	45 (30.2)	54 (36.2)	50 (33.6)
I believe that COVID-19 vaccination should be mandatory	115 (77.2)	23 (15.4)	11 (7.4)
I am sufficiently informed about the possibilities and safety of vaccination in cancer patients	96 (64.4)	28 (18.8)	25 (16.8)
I believe that cancer patients should not be vaccinated against COVID-19	44 (29.5)	73 (49.0)	32 (21.5)
